# Prevalence of dental caries and relation with nutritional status among school-age children in resource limited setting of southern Ethiopia

**DOI:** 10.1186/s12903-023-02786-6

**Published:** 2023-02-10

**Authors:** Selamawit Bassa, Shimelash Bitew Workie, Yibeltal Kassa, Dawit Worku Tegbaru

**Affiliations:** 1 Areka Town Health Office, Wolaita Zone Health Department, Wolaita Sodo, Ethiopia; 2 School of Public Health, College of Health Sciences and Medicine, Wolaita Sodo University, Wolaita Sodo, Ethiopia; 3 School of Medicine, College of Health Sciences and Medicine, Wolaita Sodo University, Wolaita Sodo, Ethiopia

**Keywords:** Dental caries, School-age children, Nutritional status, Ethiopia

## Abstract

**Background:**

Globally, dental caries appears a major public health problem and a widespread non -communicable disease. It is more prevalent among children school-age children (6–12 years), However, there are few studies that correlate dental caries with nutritional status. Thus, this study aims to determine the relation of dental caries with nutritional status among school-age children at resource limited setting of southern Ethiopia.

**Methodology:**

A community-based cross sectional study was employed on randomly selected 761 school-age children of Areka town. Data were collected by face-to-face interviewer-administered questionnaire and clinical assessment of dental caries. After that, the collected data were entered into Epi data 3.2.1 and exported to SPSS 20 for further analysis. On the other hand, bivariate and multiple logistic regression analyses were used to identify the association of dependent and independent variables. *p* Value < 0.05 was considered to declare a result as statistically significant.

**Results:**

Prevalence of dental caries among school-age children was 15.6%  (95% CI 13.0–18.5). In technical senses, 4.3% (95% CI 2.9–5.8%) of children were underweight and 14.2% (95% CI 11.7–16.6%) were overweight. However, it has been unfolded that the relationship between dental caries and nutritional status was not statistically significant with a *p* value (*p* = 0.32). Factors associated with dental caries were educational status of a mother AOR 3.14, (95% CI 1.03–9.56), not cleaning teeth AOR 7.70, (95% CI 4.00–14.85), sugared coffee drinking AOR 3.22, (95% CI 1.68–6.18.0), sweet food consumption AOR 4.19, (95% CI 1.76–9.96) and non-consuming milk AOR 5.66 ( 95% CI 1.49–21.49).

**Conclusion and recommendation:**

Dental caries at south Ethiopia were low compared to WHO’s reports on oral health on school-age children. Tooth cleaning habit, parental education, sweet food consumption and milk consumption are associated factors. Therefore, behavioral intervention on dental hygiene and dietary practices are mandatory for school-age children.

## Introduction

Oral health, as defined by the World Health Organization (WHO), is a state of being free of mouth and facial pain, oral infection and sores, periodontal (gum) disease, tooth decay, tooth loss, and other diseases and disorders that limit an individual's capacity for biting/chewing, smiling, speaking, and psychosocial wellbeing [1]. According to the 2017 Global Burden of Disease study, dental caries of the primary teeth affect more than 530 million children worldwide [2]. Consequently, dental caries is a major public health problem globally, and is the most widespread non-communicable disease (NCD) among school-age children. The study of the Global Burden of Disease during the year 2016 has ranked decay of permanent teeth in the first place among half of the world’s population (3.58 billion people), and deciduous teeth was ranked 12th position (560 million children) [3].

Most of the dental caries were untreated with considerable impacts on general health, quality of life, productivity, development, and educational performance [4]. By far, it has been proven that having good oral health enables individuals to communicate effectively, to enjoy food, to speak well, to enjoy a higher quality of life, and to have both higher self-esteem and social confidence. Tooth infection causes pain and restlessness to children resulting in decreased growth hormone and increased metabolic rate so malnutrition in children can occur [5]. More specifically, School-age children (6–12 yrs) are a top priority in oral health programs because of the high prevalence of dental caries and the development of permanent teeth in this age group [6].

The burden of dental caries in children is high. Dental caries will progress into tooth pulp and a dental abscess will result, if untreated, to loss of teeth. In advanced cases, it may interfere with dietary habits as a result affect nutritional status as well as affect sleep, work activities, and school attendance [7, 8].

Dental illnesses, particularly dental caries, cost $298 billion of the global economy in direct treatment costs, accounting for 4.6 percent of global health expenditures [9, 10]. Besides, It spends 5–10% of healthcare budgets in developed countries, and is one of the leading causes of child hospitalization in various high-income countries. It comes as no surpise that it is neglected in developing countries for, it is one of the most expensive diseases to treat [11].

Oral tissues require nourishment for development, growth, and maintenance, and oral problems can influence food choices and, ultimately, nutritional status. Prior to tooth eruption, nutritional deficiencies can affect enamel maturation and composition as well as tooth morphology and size [12–14]. Under-nutrition in children not only delays teeth development but also results a high number of carious primary teeth and exacerbates the carcinogenicity of dietary sugars. All in all, it is far beyond clear that tooth decay and early loss of teeth lead to under-nutrition [15].

The WHO set a universal goal for developing countries which should have been achieved by the year 2020. It stated that about 50% of 6 year-old children should be free of decayed teeth and the average DMFT (Decayed Missed Filled teeth) score should not be more than 3 at the age of 12 years [1]. Although dental caries and nutritional status affect one another among school-age children. There are few studies the relation to dental caries with nutritional status among school-age children. Therefore, this study was carried out to assess the prevalence of dental caries in relation to nutritional status among school-age children.

## Methods and materials

### Study setting, design and period

The sections following were conducted according to standard guidelines and procedures of observational methods. A community-based cross- sectional study design was conducted in February 2020 in Areka Town, Woliata zone, Southern Nations Nationality People’s Region (SNNPR), Ethiopia. Areka town is located in the northern part of Wolaita Zone, and is found 299 km South of Addis Ababa, 178 km from the city of Hawassa, and 29 km from Sodo (zonal capital). The town is divided into 4 kebeles with 29 urban and 18 rural villages. According to Areka town’s health office 62,254 total populations and 12,805 households and 20,083 school-age children were calculated by an indicator given from Federal ministry of Health (FMOH) for each region (32.26%) for Southern Nations Nationality Peoples’ Region (SNNPR) in 2010 [16].

### Source population and study population

The source population for this study were all school-age children (6–12 years old) living in Areka town from February–February, 2020.

### Inclusion and exclusion criteria

All school-age children permanently residing in the selected household for at least 6 months and their caregivers were included. On the other hand, severe medically ill, long-term medication, and undergoing orthodontic treatment school-age children were excluded.

### Determination of sample size

Sample size was calculated by single population proportion formula with an assumption of the prevalence of dental caries of 36.3% [17] at Gonder town. Other assumptions were 95% Confidence interval, 5% margin of error, 2 design effect, and 10% non-response rate. The total sample was **783** school-age children.

$$n= \frac{{({\mathrm{Z}}_{1-\mathrm{\alpha }/2})}^{2}*\mathrm{P}*(1-\mathrm{P}) }{{d}^{2}}$$

where Z_1-α/2_ = 95% Confidence interval (CI), P = proportion to be used (36.3%), d = margin of error (5%).

### Sampling procedure

**Fig. 1:**
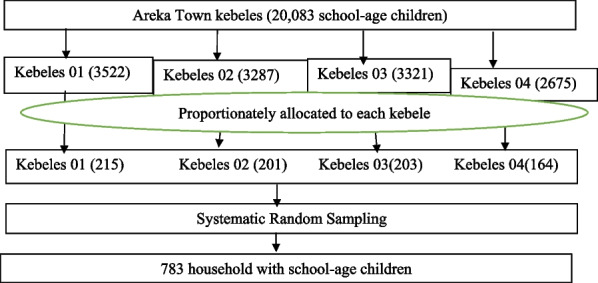
Sampling Schematic diagram showing sampling procedure for Areka town, 2020

A Systematic random sampling was employed on each kebeles of Areka town. In that regard, the sample size was proportionally allocated among all kebeles depending on their number of households. The sampling frame of households with school-age children was prepared from health posts family folder. Finally, a systematic random sampling method with k is 6th interval was applied to select the households with school-age children. The first households were selected from each kebeles randomly. If the selected the households had more than one eligible school-age child in the house, lottery method was used to select one eligible. In some scenarios, when the child was not present at home, 3 visits were made and after 3 visits, they were recorded as ‘non-response’ (Fig. 1).

### Data collection tool and procedures

The data used for this study were collected using interviewer administered questionnaire adopted from a review of literatures [17–20]. School-age children with caregivers were interviewed from the selected household. Ten BSc. graduate nurses were made to be data collectors, and two individuals with a master’s degree were made to be supervisors. Both the data collectors and supervisors were able to speak the local language. Apart from that, training was given for two days on how to collect data and supervise the data collectors. Besides, the supervisors were given additional training on oral examination of children. Besides the principal investigator was supervising during data collection.

Dental caries were assessed by clinical assessment of the oral. It was done by supervisors and the principal investigator. Dental caries was measured by the sum of Decayed, Missing and Filled Teeth (DMFT). DMFT is a tool used for measuring the prevalence of dental caries. The “D” component means Decayed tooth, “M” component is the Missing tooth, “F” component is the Filled tooth and “T” is the Tooth involved. A DMFT score above 0 indicates the presence of caries, whereas a null score indicates the absence of caries. The severity of dental caries were classified based on WHO using DMFT values, values between 0.0–1.1 very low; 1.2–2.6 low; 2.7–4.4 moderate; 4.5–6.5 high; values exceeding 6.6 will be very high [3].

The status of nutrition among children was assessed by measuring height and weight. Height was recorded with the help of a height board. Weight was measured using a Seca digital weighing scale after calibration is done. In that regard, height and weight were measured three times and took the average for further analysis. BMI score was compared to the WHO’sreference 2007 chart for age 5–18 years and assessed by using age and gender specific criteria. The following were assessed using BAZ Underweight as BAZ < − 2SD, normal ≥ − 2SD to ≤  + 1SD and overweight/ obesity as BAZ >  + 1SD [21].

Food consumption patterns were assessed using an unquantified interviewer-administered food frequency questionnaire. It was investigated by seven-day food frequency questionnaire (FFQ) consisting of 12 listed food items which are commonly used by the study population and are rich with micronutrients of interest (Calcium, Vitamin C & D), were read to the respondents who were to state the number of times they had consumed the foods in the past 7 days of the data collection period.

## Study variables

### Dependent variable

Dental caries: It was measured based on DMFT score above 0 indicates active caries and 0 indicates caries free.

### Independent variables

- **Nutritional status**: underweight, normal, overweight and obesity.
- **Socio economic and demographic variables:** age, sex, residence, grade level, parent’s education, monthly income of the family and exposure to mainstream media.
- **Dietary habits of children:** sweet consumption, meal frequency, fruit consumption and diary product consumption.
- **Oral health related behaviors:** teeth brushing**,** mouth rinsing, dental plaque, toothache, dental clinic visit and flossing habit.

### Operational definitions

- **DMFT:** Decayed (**D)**, presence of a lesion in a pit or fissure or on smooth tooth surface had a detectable softened floor, destabilized enamel or softened wall. Missing (**M)**, a person gives a history of pain and/or presence of cavity prior to extraction because of caries,. Filled (**F)** presence of one or more restorations.
- **Dental caries: A** DMFT score above 0 indicates the presence of caries, whereas a null score indicates the absence of caries.
- **Regular tooth brushing-** reported of brushing tooth with toothbrush and fluoride toothpaste twice a day or after every meal for about two minutes.
- **Flossing habit-** is removing of food and dental plaque from space between two teeth areas a toothbrush is unable to reach.

### Data quality assurance

The Questionnaire was first prepared by English language. Then, it was translated to local language Wolayitatoo by local and English language experts. After that, it was translated back to English by different language experts to maintain its consistency. Pre-test was conducted by taking 5% of the sample which is 39 school-age children in Bombe town outside the study area after training given. This verified the clarity of instruments and helped to familiarize data collectors to the instrument and necessary correction was made accordingly. The supervisors and the principal investigator made daily checkup on the data collection process to ensure the completeness and consistency. Any error found during the process was corrected immediately.

### Data management and analysis procedures

Data were entered into Epi data version 3.2.1 after ensuring completeness of each questionnaire. After that, it was exported to SPSS version 20 for further analyses. Descriptive statistics was conducted to determine the frequencies and percentages of different variables. Bivariate and multiple logistic regression analyses were done to identify variables that were associated with the dependent variables. Independent variables having *P* value less than 0.25 on bivariate analysis were nominated for multiple logistic regression analysis for further confounding effect control [22]. Hosmer and Lemeshow goodness of fit test was used to assess the fitness of the model during multiple logistic regression analyses. Normality was checked by Kolmogorov smirnov test of normality.

Crude and adjusted odds ratios together with their corresponding 95% confidence intervals were computed and interpreted accordingly. *P* value < 0.05 was considered to declare a result as statistically significant. A correlational statistical test was used to determine the relation between nutritional status and caries occurrence. BMI- for-age (BAZ) was used to assess each child's nutritional status. BMI- for-age (BAZ) score parameter was calculated using WHO anthroplus 2007 version 3.2.2 software.

## Result

### Socio demographic characteristics of respondents

A total of 761 participants were included in the analysis, resulting in a response rate of 97.2%. Out of these, three hundred thirty-one (43.5%) school-aged children were between the ages of 6 and 8 years, with a median age of 9.0 years and an interquartile range (IQR) of 4, and 54 percent of them were girls. Most of the respondents, 723 (95%) were urban residents. Five hundred twelve (67.3%) of them were attending class at governmental school, and they were 0–4 class students. On the other side, two hundred forty-four (32.1%) mothers or caregivers had no formal education, but 308(40.5%) of fathers were college graduates. Above half of (52.7%) the mothers’ or caregivers’ occupation was housewife, and 296 (38.9%) fathers were government employees. It has been seen that the majority of the households 505 (66.4%) had exposure to the mainstream meadia (TV) (Table 1).

**Table 1:** Frequency distributions of socio-demographic characteristics of children at Areka town, Woliata zone 2020 (n = 761)

Variables		Frequency	Percentages (%)
Residence	Urban	723	95
	Rural	38	5
Sex	Male	350	46
	Female	411	54
Age (years)	6–8	331	43.5
	9–10	218	28.6
	11–12	212	27.9
School type	Governmental	512	67.3
	Private	249	32.7
Grade level of child	0–4	644	84.6
	5–8	117	15.4
Educational status of mother	No formal education	244	32.1
	Primary education	192	25.2
	Secondary education	212	27.9
	College and above	113	14.8
Educational status of father	No formal education	83	10.9
	Primary education	89	11.7
	Secondary education	281	36.9
	College and above	308	40.5
Occupation of the mother	Employee	108	14.2
	House wife	401	52.7
	Merchant	248	32.6
	Other	4	0.5
Occupation of the father	Government Employee	296	38.9
	Merchant	296	38.9
	Farmer	48	6.3
	Laborer	112	14.7
	Other	9	1.2
TV ownership	Yes	505	66.4
	No	256	33.6
Time on TV screen per day (n = 505)	1–2 h	219	43.4
	> 2 h	286	56.6

### Oral health-related behaviors of children

It has been found that the majority of the children, (72.5%), never clean their teeth; of those who do, 416 (75.3%) clean their teeth once or less than once a day, while just 136 (24.7%) clean them on a regular basis. Two hundred and forty-nine children (45.1%) used mouthwash/rinse with water, whereas 209 people (37.9%) used paste-based toothbrushes. Almost half 288 (52.2%) clean their teeth in the morning before breakfast. On the other hand, two hundred seventeen (39.3%) children spent only one minute on teeth cleaning. Almost all 372 (96.1%) of the children who had a flossing habit used a conventional tool, and 193 (49.9%) flossed when something entered their teeth. The majority of the participant 467(61.4%) don’t know when to renew toothbrush. The majority of them (92.6%) have never visited a dental clinic, and of those who have, the majority went for emergency dental pain treatment 52 (41.4%) and extraction 5 (8.6%) (Table 2).

**Table 2:** Oral health–related behaviors of children at Areka town, Woliata zone 2020

Variables		Frequency	Percentages (%)
Ever cleaning teeth (n = 761)	Yes	552	72.5
	No	209	27.5
Frequency of teeth cleaning in a day (n = 552)	≤ 1 daily	416	75.3
	≥ 2 daily	136	24.7
Way of cleaning teeth (n = 552)	Toothbrush with paste	209	37.9
	Toothbrush without paste	24	4.3
	Mouthwash / rinse with water	249	45.1
	Mefakia (twig brush)	70	12.7
Time of tooth cleaning (n = 552)	Morning before breakfast	288	52.2
	Morning after breakfast	138	25
	Noon before lunch	10	1.8
	After each feeding	13	2.4
	Whenever remember	74	13.4
	When think teeth is dirty	26	4.7
	Others	3	0.5
Time spent teeth cleaning (n = 552)	Less than one minute	153	27.7
	One minute	217	39.3
	Two minutes	121	21.9
	More than two minutes	61	11.1
Flossing practice (n = 761)	Yes	387	50.9
	No	374	49.1
Tools use for flossing (n = 387)	Professional	15	3.9
	Traditional	372	96.1
Time of flossing (n = 378)	Regularly	64	16.5
	Something enters in to teeth	193	49.9
	When feel should	130	33.6
Time of toothbrush renewed (n = 294)	Every 3 month	182	61.9
	Every 6 month	76	25.9
	Every 1 year	32	10.9
	Other	4	1.4
Tooth cleaning helps in preventing caries (n = 761)	Yes	640	84.1
	No	68	8.9
	Don’t know	53	7.0
Dental clinic visit during the past 1 year (n = 761)	Yes	58	7.6
	No	703	92.6
Frequency of dental visit (n = 58)	Regularly every 6–12 months	3	5.2
	Occasionally	31	53.4
	When have dental pain	24	41.4
Reason for visiting dentist (n = 58)	Dental pain	52	89.6
	For extraction	5	8.6
	For tooth cleaning	1	1.73
Dental pain/toothache history (n = 761)	Yes	55	7.2
	No	706	92.8

### Dietary habits of children

More than three forth of the children 590 (77.5%) drink tea with an added sugar. And two hundred twenty-one (29%) of the children drink coffee with an added sugar. Besides, three hundred nineteen (41%) of the children are found to drink soft drinks and two third of the children 553 (70%) consume sweets. Among these sweet food consumers, 272 (51.0%) consume less than once per week and 141(26.5%) consume on a daily basis. Five hundred sixty-five (74.2%) of the children consume milk less than once per week (Table 3).

**Table 3:** Dietary habits of the children at Areka town, Woliata zone 2020

Variables		Frequency (n = 761)	Percentages (%)
Consumption of sugared tea	Yes	590	77.5
	No	171	22.5
Number of cups of sugared tea per day (n = 590)	1–2	562	95.3
	> 2	28	4.7
Consumption of sugared coffee	Yes	221	29
	No	540	71
Consumption of soft drinks	Yes	319	41.9
	No	442	58.1
Number of bottles of soft drink per day	1–2	290	90.9
	> 2	29	9.1
Consumption of sweet foods	Yes	553	70
	No	228	30
Frequency of sweet foods per month (n = 553)	Daily	141	26.5
	2-3 days in a week	98	18.4
	Once a week	22	4.1
	Less than once per week	272	51
Consumption of milk per month	No, do not	47	6.2
	Less than once per week	565	74.2
	Usually /daily to weekly/	149	19.6

### Consumption frequency of selected foods and beverages in the past 7 days

The highest percentage among leafy green vegetables, banana, wheat, and maize to be taken at least once in the last seven days with a frequency rate of 759 (99.7%), 756 (99.3%), 31 (96.1%), and 727 (95.5%) respectively. It shows abundant consumption of vitamin C whereas fish and fish oil, meat and orange had been consumed at least once by 24 (3.2%), 370 (40.9%) and 460 (60.4%) respectively, and these are the lowest percentage observed and shows lacks calcium and vitamin D (Table 4).

**Table 4:** Consumption frequency of selected foods in the 7 days preceding the study of the children in Areka town, Woliata zone 2020

Variable	Categories	Frequency	Percentage
Milk and milk product	Yes	699	91.9
	No	62	8.1
Fish and fish oil	Yes	24	3.2
	No	737	96.8
Meat	Yes	370	40.9
	No	391	59.1
Egg	Yes	661	86.9
	No	100	13.1
Leafy green vegetable	Yes	759	99.7
	No	2	0.3
Banana	Yes	756	99.3
	No	5	0.7
Orange	Yes	460	60.4
	No	301	39.6
Beans	Yes	702	92.2
	No	59	7.8
Peas	Yes	648	85.2
	No	113	14.8
Maize	Yes	727	95.5
	No	34	4.5
Barly	Yes	499	65.6
	No	262	34.4
Wheat	Yes	731	96.1
	No	30	3.9

### Nutritional status and prevalence of dental caries

The median height and weight were 130.00 cm with IQR 16.00 and 29.00 kg with IQR 9 respectively. Among all children, 33 (4.3%) were underweight based on BAZ (< − 2 SD) with (95% CI 2.9–5.8), 620 (81.5%) were within the normal range (**≥ **− 2 SD and ≤  + 1 SD) with (95% CI 78.7–84.4) and 108 (14.2%) were overweight/obese (> + 1 SD) with (95% CI 11.7.0–16.6). The median score of BAZ was 0.37 with an IQR of 1.14.

The prevalence of dental caries was 119 (15.6%) with (95% CI 13.0–18.5). The prevalence is almost equally distributed among males 53 (15.1%) and females 66 (16.1%). The score of DMFT ranged from 0 to 8 and the mean DMFT was 0.27 (± 0.75). The prevalence of dental caries was higher among children who were underweight and overweight.21.2% and 18.5% respectively. On the other hand, it decreased among children with normal weight 14.8%; it was checked by the chi-square test, and was statistically not significant (Table 5).

**Table 5:** Prevalence of dental caries according to BAZ score school-age children at in Areka town, Woliata zone, 2020

BAZ	Caries free		Caries active		Total		*P* value
	No	Percent	No	Percent	No	Percent	
underweight	26	78.8	7	21.2	33	4.3	0.32
Normal	528	85.2	92	14.8	620	81.5	
Overweight/obese	88	81.5	20	18.5	108	14.2	
Total	642	84.4	119	15.6	761	100	

### Relationship of dental caries with nutritional status

The relationship between dental caries and nutritional status was not statistically significant with a *P* value (*P* = 0.32). The DMFT score was positively correlated to BAZ score, but the relationship did not show strong relationship with correlation coefficient value (r = 0.011).

### Factors associated with dental caries

Place of residence, school type, educational status of the mother, exposure to the media, cleaning teeth, consumption of sugared coffee, sweet foods consumption, and milk consumption were candidate variables for multiple logistic analysis at *P* value < 0.25. In that regard, the multiple logistic regression analysis revealed the educational status of the mother, habit of cleaning teeth, consumption of sugared coffee, sweet foods consumption, and milk consumption had a statistical association.

Children whose mothers were not educated were 3.14 times (95% CI 1.03–9.56) at risk as compared to those whose mothers are educated. Besides, children who did not clean their teeth were 7.70 times more likely to have caries as compared to those who had cleaned their teeth (AOR = 7.70, 95% CI 4.00–14.85). On the other hand, children who had not consumed sugared coffee were 3.22 times reduced risk to have caries as compared to those who consume sugared coffee (AOR = 3.22, 95% CI 1.68–6.18). Meanwhile, children who had consumed sweet foods were 4.19 times at high risk as compared to those who had not consumed sweet foods (AOR = 4.19, 95% CI 1.76–9.96). Children who had not to consumed milk were 5.66 times more likely to have dental caries than those who had consume milk usually (AOR = 5.66, 95% CI 1.49–21.49). There was no statistically significant association between dental caries and nutritional status of the children (Table 6).

**Table 6:** Multiple logistic regression analysis for factors associated with dental caries in Areka town, Woliata zone 2020

Variables	With dental caries	Dental caries free	COR	AOR
Educational status of mother				
No formal education	49 (20.1%)	195 (79.9%)	5.42 (2.10–14.03)	3.14 (1.03–9.56) *
Primary education	36 (18.8%)	156 (81.2%)	4.98 (1.89–13.11)	2.67 (0.84–8.50)
Secondary education	29 (13.7%)	183 (86.3%)	3.42 (1.28–9.10)	2.30 (0.78–6.77)
College and above	5 (4.4%)	108 (95.6%)	1	1
Cleaning teeth				
Yes	50 (9.1%)	502 (90.9%)	1	1
No	69 (33.0%)	140 (79.6%)	4.94 (3.28–7.45)	7.70 (4.00–14.85) ***
Sugared coffee consumption				
Yes	45 (20.4%)	176 (55.2%)	1.61 (1.7–2.42)	3.22 (1.68–6.18) ***
No	74 (13.7%)	466 (86.3%)	1	1
Sweet foods consumption				
Yes	100 (18.8%)	433 (81.2%)	2.54 (1.51–4.26)	4.19 (1.76–9.96) ***
No	19 (8.3%)	209 (91.7%)	1	1
Milk consumption				
Don’t consume	15 (31.9%)	32 (68.1%)		5.66 (1.49–21.49) **
Occasionally	84 (14.9%)	481 (85.1%)		1.27 (0.60–2.71)
Usually ( daily to weekly)	20 (13.4%)	129 (86.6%)	1	1
Nutritional status				
Underweight	7 (21.2%)	26 (78.8%)	1	1
Normal	92 (14.8%)	528 (85.2%)	0.64 (0.27–1.53)	0.43 (0.12–1.48)
Overweight/obese	20 (18.5%)	88 (81.5%)	0.84 (0.32–2.21)	1.02 (0.26–4.00)

^*^P < 0.05, **P < 0.01, ***P < 0.001

## Discussion

The prevalence of dental caries was found 15.6% in line with the study conducted in Bahir Dar city, Ethiopia (21.8%) [18], Tanzania (17.6%) [23], and Nigeria (21.7%)[24]. However, it was lower than studies carried out in other parts of Ethiopia (36.3–48.5%) [17–20], Eritrea (78%) [25], and India (76.25%) [26]. It was also much lower than the figures reported by the WHO on oral health among of school children (60–90%) [7]. These figures could be due to socio-demographic and economic characteristics, and dietary behaviors. This might be due to the fact that 95% of the study participants were from urban residences.

Dental caries, on the other hand, was associated with the educational status of the mother, cleaning teeth, sugared coffee consumption, sweet consumption, and milk consumption. Educational status of the children’s mothers was found to be significantly associated with dental caries. Hence, there follows a simple logic here: as the education level of the mother increases, the probability of dental caries getting reduces. This was supported by similar findings from other parts of Ethiopia [17, 19]. It might be the fact that mothers who were educated are more likely to gain knowledge about dental health. It also gives an opportunity to have increased access to information from different media.

Dental caries development and teeth brushing practices had an inverse relationship. This goes in line with the study conducted in different parts of Ethiopia; Bahir Dar, Gonder, Adiss Abeba, and Finote Selam [17–20]. This finding is also in line with the study conducted in Tamil Nadu India [27]. It is generally true that cleaning teeth will remove away the food debris from the oral cavity. Therefore, *Streptococcus mutans* cannot get enough nutrients and time for growth, and there will be no acid production that causes dental caries development.

In this study, those children who had consumed sugared coffee do have an increased the chance of developing caries than those who had not consumed. Sweet consumption was also significantly associated with dental caries. Children who consumed more sweet foods frequently had a higher chance of developing dental caries than those who consumed sweet foods rarely. This is supported by a study done in Adiss Abeba, Finote Selam, and Harar town [19, 20, 28]. It is also in line with a study done in Mbeya City, Tanzania [23]. This might be related to much acid production by microorganisms like bacteria to teeth because of fermentation of the sweet foods. Gradually, the enamel of the tooth goes into dental caries [29].

Children who consume milk on a regular basis were 5.66 times safer than those children not consuming milk. It might be Cow milk had small amount of fluoride concentration and was similar to other studies [30].

Nutritional status (BAZ) and dental caries development was not significantly associated. This is in line with a study done in India (14). Dental caries is a multifactorial disease with several identified risk factors however the correlation between dental caries and nutritional status is not well understood, and requires further experimental research.

Difficulties in using radiological examination at the field level might reduce the actual magnitude of dental caries was the limitation of the study. All limitation of cross-sectional design was the limitations of this study.

## Conclusion and recommendations

The prevalence of dental caries was found to be low compared to the WHO’s report on oral health among school children. The educational status of mothers, habit of cleaning teeth, consumption of sugared coffee, consumption sweet food, and milk consumption were the associated factors for dental caries. There was no significant association between dental caries and nutritional status. Behavioral intervention on dental hygiene and dietary habits should be given to children and parents. Moreover, further studies using all methods of diagnosis of dental caries and assessment of knowledge, attitude, and practices of children and their parents on dental hygiene should be recommended.

## Data Availability

The datasets during and/or analyzed during the current study is available from the corresponding author on reasonable request.

## Abbreviations

AOR: Adjusted odds ratio
BAZ: BMI for age Z score
BMI: Body Mass Index
CI: Confidence interval
COR: Crude odds ratio
DMFT: Decayed missed filled teeth
IQR: Interquartile range
GBD: Global burden of disease
NCD: Non communicable disease
SD: Standard deviation
SNNPR: Southern Nations Nationality People Region
WHO: World Health Organization
